# Oxidative–Nitrosative Stress and Routine Biochemical Parameters in Amyotrophic Lateral Sclerosis: Associations with Clinical Status and Disease Duration—A Pilot Study

**DOI:** 10.3390/biom16050721

**Published:** 2026-05-13

**Authors:** Pavlína Malá, Nela Váňová, Ondřej Malý, Oldřich Vyšata

**Affiliations:** 1 Department of Neurology, University Hospital Hradec Králové, 500 05 Hradec Králové, Czech Republic; oldrich.vysata@fnhk.cz; 2 Department of Neurology, Faculty of Medicine in Hradec Králové, Charles University, 500 03 Hradec Králové, Czech Republic; 3 Department of Pharmaceutical Chemistry and Pharmaceutical Analysis, Faculty of Pharmacy in Hradec Králové, Charles University, 500 05 Hradec Králové, Czech Republic; vanovan@faf.cuni.cz; 4 Military Faculty of Medicine, University of Defense, 500 01 Hradec Králové, Czech Republic; ondrej.maly@fnhk.cz; 5 Department of Surgery, University Hospital Hradec Králové, 500 05 Hradec Králové, Czech Republic

**Keywords:** amyotrophic lateral sclerosis, motor neuron disease, neurodegeneration, disease duration, oxidative–nitrosative stress, 3–nitrotyrosine, non-protein thiols, oxidative–nitrosative stress biomarkers, plasma biomarkers

## Abstract

Background: This pilot study examined whether oxidative–nitrosative stress is associated with clinical status in amyotrophic lateral sclerosis (ALS). We analyzed associations between plasma markers of oxidative–nitrosative imbalance and ALSFRS–R, disease duration, survival, and routine biochemical parameters. Methods: Twenty-nine ALS patients fulfilling the Gold Coast diagnostic criteria were enrolled. Plasma levels of 3-nitrotyrosine (3–NT), 8-oxo-2′-deoxyguanosine (8–oxodG), malondialdehyde (MDA), glutathione (GSH), non-protein thiols (NP–SH), and non-protein disulfides (NP–SS–NP), as well as creatinine, urea, uric acid and BMI, were measured. Associations with ALSFRS–R and disease duration were evaluated using non-parametric correlation analyses and second-order polynomial regression (adjusted R^2^), while survival was explored using Kaplan–Meier analysis and multivariable Cox regression. Given the modest sample, we considered statistical power and applied Benjamini–Hochberg false discovery rate (FDR) correction within marker families. Results: At the uncorrected significance level, 3–NT showed a positive correlation with ALSFRS–R and a negative correlation with disease duration, and NP–SH correlated negatively with disease duration; however, these associations did not remain significant after FDR correction (FDR-adjusted *p* ≥ 0.099). Other oxidative–nitrosative markers and biochemical parameters showed no robust relationships with clinical measures. In Cox models, 3–NT was not significantly associated with survival (HR 3.44 per 1 nM, 95% CI 0.25–47.97, *p* = 0.358), whereas older age predicted higher mortality (HR 1.05 per year, 95% CI 1.00–1.10, *p* = 0.036). Conclusions: 3–NT and NP–SH exhibited the strongest trends among the investigated markers, but their clinical associations in this small cross-sectional cohort remain exploratory and require confirmation in larger longitudinal studies.

## 1. Introduction

Amyotrophic lateral sclerosis (ALS) is a heterogeneous neurodegenerative syndrome characterized by progressive degeneration of both upper motor neurons (projecting from the motor cortex to the brainstem and spinal cord) and lower motor neurons (projecting from the brainstem or spinal cord to skeletal muscle), leading to a combination of motor and extra-motor manifestations [1].

In 5–15% of individuals with ALS, either ALS or frontotemporal dementia occurs in other family members, indicating a familial form of the disease [2,3,4]. In such families, a single pathogenic variant is usually considered sufficient to cause ALS, and to date, roughly 30 genes have been implicated in familial ALS, encompassing diverse biological pathways and mechanisms [5].

Most patients, however, have no family history of ALS, and in these cases, the disease is considered sporadic and thought to arise from a combination of environmental and genetic risk factors [6]. Numerous genetic susceptibility factors for sporadic ALS have now been identified, whereas efforts to define consistent environmental contributors have been considerably less successful [1].

Despite extensive research, the initial trigger of the neurodegenerative process remains unclear. It is assumed that a combination of genetic predisposition and environmental factors initiates pathological mechanisms that subsequently spread from one motor region to another, likely through the interaction of misfolded proteins, inflammatory responses, and disturbances of cellular homeostasis [7,8]. It has been hypothesized that this as-yet unidentified initiating factor may be linked to the early onset of oxidative–nitrosative stress, which plays a central role in initiating motor neuron injury [9].

During disease progression, several interrelated pathophysiological processes have been consistently observed. These include mitochondrial dysfunction [10], glutamate-induced excitotoxicity, activation of microglia and astrocytes [11], impairment of axonal transport, and the previously mentioned oxidative–nitrosative stress resulting from a disrupted balance between reactive oxygen species (ROS) and reactive nitrogen species (RNS) [12,13]. These molecules can damage lipids, proteins, and DNA, thereby contributing to neuronal apoptosis and disease progression [14].

Under physiological conditions, reactive oxygen species (ROS) and reactive nitrogen species (RNS) are continuously generated in human cells and act as signaling mediators in numerous cellular processes [15]. However, when their production exceeds the capacity of antioxidant defense systems, or when these systems fail, a state known as oxidative–nitrosative stress arises. Because these processes often occur simultaneously and are closely interconnected, the term oxidative–nitrosative stress is commonly used to describe their combined effects [16].

Both types of stress lead to structural and functional cellular damage through oxidation and nitration of proteins, lipids, and DNA. Oxidative stress is associated, for example, with lipid peroxidation or oxidation of guanine residues in DNA [17,18]. Nitrosative stress, on the other hand, is typically characterized by nitration of tyrosine residues in proteins, forming 3–nitrotyrosine (3–NT)—a stable and widely used in vivo marker of nitrosative stress [19].

Although the underlying mechanisms differ, oxidative–nitrosative stress shares several key features: both are closely linked to inflammatory responses and contribute to neuronal apoptosis and necroptosis [16,20]. In the context of ALS, these forms of stress have been repeatedly shown to play a role not only in early motor neuron injury but also in disease progression [14,21,22].

Several clinical studies have provided direct evidence of oxidative–nitrosative imbalance in ALS. Baillet et al. reported altered levels of multiple oxidative stress markers in patients with ALS compared with healthy controls, supporting the presence of systemic redox dysregulation [23].

In the large multicenter ALS COSMOS study, baseline plasma creatinine and uric acid were associated with functional status and survival, and longitudinal analyses showed declining plasma creatinine and uric acid alongside increasing urinary oxidative stress markers, with creatinine emerging as a particularly robust predictor of outcome [24].

Further evidence of the role of oxidative–nitrosative stress in ALS pathogenesis was provided by Nagase et al., who found an unfavorable redox profile in patients with ALS, including increased oxidized coenzyme Q10, reduced uric acid levels and altered fatty acid composition. Treatment with edaravone was associated with a rise in uric acid and a slower decline in ALSFRS–R scores, further supporting the therapeutic rationale for targeting oxidative stress in ALS [25].

The aim of this pilot study was to expand the current understanding of oxidative–nitrosative stress in ALS pathogenesis. Unlike most previous studies, which focused on isolated biomarkers or narrow clinical correlations, we assessed a broader panel of plasma markers of oxidative–nitrosative imbalance and examined their associations with functional status, disease duration and survival. Based on these considerations, we formulated the following three main hypotheses: (1) the levels of selected oxidative–nitrosative stress markers correlate with the functional status of ALS patients as measured by ALSFRS–R; (2) the levels of these markers correlate with disease duration at the time of sampling; and (3) the levels of these markers are associated with overall survival in ALS patients. In addition, we evaluated whether these biomarkers could potentially serve as prognostic indicators or tools for monitoring the effects of targeted antioxidant therapy and explored their relationships with common biochemical parameters, which may indirectly reflect systemic redox imbalance and the overall physiological status of ALS patients.

Creatinine, a metabolic byproduct of muscle tissue, is widely used as an indirect indicator of muscle mass. In ALS, it can therefore reasonably be assumed that progressive muscle atrophy is accompanied by a gradual decline in creatinine concentrations over time. In a large prospective cohort of patients with ALS, lower baseline creatinine and a faster decline in creatinine over time were independently associated with poorer functional status and shorter survival, supporting serum creatinine as a simple and accessible prognostic marker in this disease [26].

Skeletal muscles are also a major source of antioxidant enzymes such as superoxide dismutase (SOD) and glutathione peroxidase [27]. On this basis, we propose the following pathophysiological concept: reduced muscle mass is expected to result not only in lower creatinine production but also in generally diminished systemic antioxidant capacity. Under conditions of pronounced oxidative–nitrosative stress, this may further accelerate muscle degradation, such that a higher oxidative burden is associated with progressive loss of muscle tissue and, consequently, lower creatinine levels. In line with this rationale, we hypothesize an inverse association between plasma 3–nitrotyrosine (3–NT), a marker of nitrosative stress and creatinine concentrations.

Uric acid is a major endogenous antioxidant capable of neutralizing reactive species, including peroxynitrite, thereby limiting the nitration of tyrosine residues [28]. Lower uric acid levels may thus reflect depletion of antioxidant defenses and an increased risk of oxidative damage, including elevated 3–NT formation. Moreover, some studies have suggested that reduced uric acid levels are associated with poorer prognosis in ALS patients [29,30].

Urea, as the product of amino acid catabolism, may, in the context of oxidative stress, reflect enhanced protein breakdown—for instance, due to oxidative–nitrosative damage to structural or enzymatic proteins. Increased urea formation could therefore represent an indirect marker of oxidative–nitrosative tissue injury, including muscle tissue damage.

Based on these considerations, we formulated a set of secondary hypotheses testing the relationships between nitrosative stress markers (particularly 3–NT) and selected biochemical parameters. Specifically, we hypothesized that plasma 3–NT would be positively correlated with urea and negatively correlated with creatinine and uric acid, that creatinine would correlate positively with ALSFRS–R score and survival duration, that lower uric acid concentrations would be associated with shorter survival, and that higher urea concentrations would be associated with shorter survival.

## 2. Materials and Methods

### 2.1. Study Population

A total of 29 patients with amyotrophic lateral sclerosis (ALS) were enrolled in the study, all meeting the Gold Coast diagnostic criteria. Of these, 22 were men (75.9%) and 7 were women (24.1%). The mean age of the cohort was 61.0 ± 14.8 years (range 34–87 years). Functional impairment, assessed using the ALS Functional Rating Scale-Revised (ALSFRS–R), had a mean score of 34.5 ± 8.6 points (range 10–45). All participants were non-smokers and had normal renal function, as assessed by serum creatinine within the reference range. Age, sex, BMI and serum creatinine were later included as covariates in the Cox regression model. Furthermore, all patients were treated with riluzole according to standard clinical practice.

The mean body mass index (BMI) was 24.6 ± 4.7 kg/m^2^ (range 16.9–38.8), corresponding predominantly to normal weight or mild overweight. The mean survival time from diagnosis was 19.6 ± 14.5 months (range 0–43 months; calculated only for patients with a known date of death). The mean disease duration at the time of biological sampling was 19.5 ± 12.4 months (range 3–55 months, exceptionally up to 110 months).

Overall, the cohort consisted mainly of older male patients with heterogeneous degrees of functional impairment, variable body weight, and wide variation in both survival and disease duration, reflecting the well-known clinical heterogeneity of ALS.

### 2.2. Analytical Methods

The following oxidative stress markers were determined in plasma samples:

- 3–nitrotyrosine (3–NT)—a marker of oxidative protein damage,
- 8-hydroxy-2′-deoxyguanosine (8–oxodG)—a marker of DNA oxidation,
- malondialdehyde (MDA)—a product of lipid peroxidation,
- reduced glutathione (GSH),
- non-protein thiols (NP–SH), and
- non-protein disulfides (NP–SS–NP)—markers of antioxidant capacity.

The biomarker panel was selected to capture complementary domains of oxidative–nitrosative imbalance. 3–nitrotyrosine (3–NT) was used as a marker of protein nitration and nitrosative stress, 8-oxo-2′-deoxyguanosine (8–oxodG) as a marker of oxidative DNA damage, and malondialdehyde (MDA) as an index of lipid peroxidation. Reduced glutathione (GSH), non-protein thiols (NP–SH), and non-protein disulfides (NP–SS–NP) were included to reflect systemic antioxidant capacity and thiol redox balance.

Validated chromatographic methods, based on previously published protocols, were employed for the quantification of oxidative–nitrosative stress biomarkers in plasma samples [31,32].

MDA was determined after hydrolysis of protein-bound MDA, derivatization with 2,4-dinitrophenylhydrazine (DNPH) and purification by solid-phase extraction on C18 cartridges. Quantification was carried out by high-performance liquid chromatography coupled with tandem mass spectrometry and atmospheric pressure chemical ionization (HPLC-MS/MS with APCI), using deuterated d_2_-MDA as an internal standard and a multi-point calibration curve.

GSH and NP–SH were quantified after derivatization with 5,5′-dithiobis-(2-nitrobenzoic acid) (DTNB, Ellman’s reagent) and subsequent analysis by high-performance liquid chromatography with ultraviolet detection (HPLC-UV) on a C18 column. NP–SS–NP were reduced with sodium borohydride (NaBH_4_), derivatized with DTNB and analyzed under the same chromatographic conditions, with external calibration for both reduced and oxidized forms.

For 3–NT and 8–oxodG, plasma samples were spiked with stable-isotope-labeled internal standards and pre-treated by solid-phase extraction (SPE) on polymeric cartridges. The analytes were then separated by ultra-high-performance liquid chromatography (UHPLC) on a polar C18 column and quantified using tandem mass spectrometry with electrospray ionization (UHPLC-MS/MS with ESI) in selected reaction monitoring (SRM) mode, employing multipoint calibration curves in plasma matrix.

### 2.3. Statistical Analysis

Continuous variables are presented as mean ± standard deviation or median (interquartile range), as appropriate, and categorical variables as counts and percentages. Because of the limited sample size and the absence of strong normality assumptions, associations between oxidative–nitrosative markers, clinical parameters (ALSFRS–R, disease duration, survival) and routine biochemical variables were primarily evaluated using Spearman’s rank correlation coefficient and Kendall’s tau.

To explore potential curvilinear relationships, we additionally fitted second-order polynomial regression models with the respective oxidative–nitrosative marker as the dependent variable and ALSFRS–R or disease duration as independent variables. For each model, we report the adjusted coefficient of determination (adjusted R^2^) and the *p*-value for the quadratic term. When the quadratic term was not statistically significant and residual diagnostics did not indicate systematic deviations from linearity, interpretation was based primarily on the monotonic correlation analyses rather than on non-linear effects.

Given the exploratory nature of this pilot study, the main outcomes were defined a priori as the associations between plasma 3–NT and ALSFRS–R and between 3–NT and disease duration. All other tests, including analyses of additional oxidative–nitrosative markers, biochemical parameters and survival, were considered secondary and hypothesis-generating. Within each family of related tests (e.g., six oxidative–nitrosative markers vs. ALSFRS–R; six markers vs. disease duration), we applied a Benjamini–Hochberg false discovery rate (FDR) correction and report both unadjusted and FDR-adjusted *p*-values. For key correlations, we additionally provide 95% confidence intervals for Spearman’s rho.

Although the sample size was constrained by feasibility, an a priori power consideration was performed; the study has reasonable power to detect moderate correlations (e.g., r ≈ 0.45–0.50) at α = 0.05 but limited power for weaker effects. Post hoc power estimates for the observed correlations confirmed the limited precision of effect size estimates, underscoring the pilot character of our analyses. All statistical tests were two-tailed, and a *p*-value < 0.05 was considered statistically significant before correction; FDR-adjusted *p*-values are provided to account for multiple testing. Statistical analyses were performed in Python using NumPy (version 1.26.4) for numerical operations, SciPy (version 1.13.1) for non-parametric correlations and hypothesis testing, and statsmodels (version 0.14.4) for regression modeling and the estimation of confidence intervals. Kaplan–Meier survival curves were generated using the Numiqo online tool (numiqo.com), while Cox proportional hazards regression and related inferential statistics were conducted in Python.

### 2.4. Survival Analysis

Overall survival was defined as the time from ALS diagnosis to death from any cause; patients who were alive at the last documented follow-up were censored at that date. For patients with unknown survival status, survival time was either censored at the last available clinical contact or, when such information was not available, excluded from the survival analysis (details provided in Table 3). Baseline plasma levels of oxidative–nitrosative markers and routine biochemical parameters were related to survival using Kaplan–Meier curves and Cox proportional hazards regression.

For Kaplan–Meier analyses, patients were stratified into “high” versus “low” groups according to the median baseline 3–NT concentration (and analogously for other markers in exploratory analyses). Survival curves were compared using the log-rank test. In Cox models, biomarkers were entered as continuous predictors (per unit increase), and hazard ratios (HRs) with 95% confidence intervals (CIs) were reported. Given the limited number of deaths, we restricted multivariable Cox models to a small set of clinically relevant covariates. The main model included baseline 3–NT together with age, sex and BMI as predictors to obtain an adjusted estimate of the association between 3–NT and survival, while acknowledging the exploratory nature and limited precision of these estimates. All survival analyses were considered exploratory and are primarily intended to provide preliminary effect size estimates for future studies.

## 3. Results

### 3.1. Correlation Between Selected Oxidative–Nitrosative Stress Markers and Functional Status Assessed by ALSFRS–R

#### Interpretation of Combined Results

**Figure 1:**
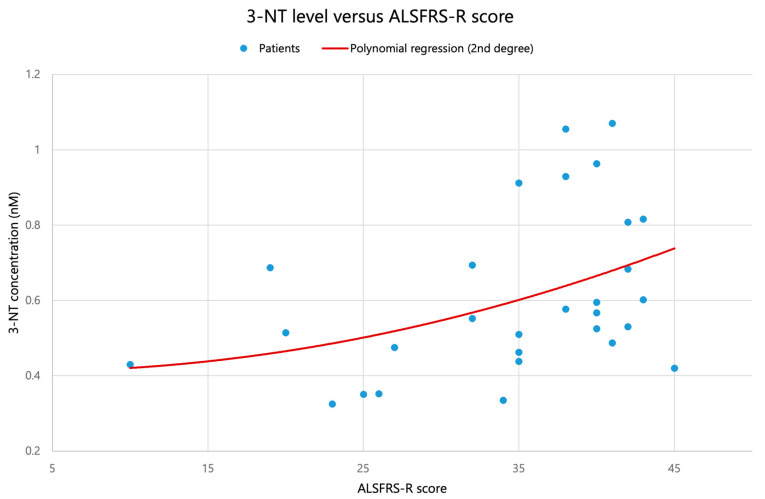
Association between plasma 3–nitrotyrosine (3–NT) concentration and functional status (ALSFRS–R) in 29 patients with ALS. Points represent individual patients. The solid line illustrates the fitted trend and is shown for visualization of the observed relationship; statistical interpretation is based primarily on Spearman’s rank correlation because of the limited sample size and non-normal distribution assumptions.

A statistically significant relationship was observed for 3-nitrotyrosine (3–NT) at the uncorrected significance level (Spearman ρ = 0.418, *p* = 0.024; Kendall τ = 0.311, *p* = 0.020), see Figure 1. However, after applying Benjamini–Hochberg false discovery rate correction for multiple testing across six markers, this association did not reach the adjusted significance threshold (FDR-adjusted *p* = 0.144). The moderate positive correlation nevertheless suggests a potential relationship worthy of confirmation in larger studies. For details, see Table 1.

For the other oxidative–nitrosative stress markers, no statistically significant relationships with ALSFRS–R were found—neither linear nor nonlinear. Spearman’s and Kendall’s correlation coefficients, as well as the coefficients of determination (R^2^) and *p*-values for the quadratic terms, consistently exceeded the threshold for statistical significance (*p* > 0.05).

The highest R^2^ value was observed for non-protein disulfides (NP–SS–NP [µM]) (R^2^ = 0.202), although this relationship with ALSFRS–R also failed to reach statistical significance in any of the applied analyses.

Among the oxidative–nitrosative stress markers examined, 3-nitrotyrosine (3–NT) was the only marker that reached statistical significance at the uncorrected level in its correlation with functional status as assessed by ALSFRS–R (Spearman ρ = 0.418, *p* = 0.024; Kendall τ = 0.311, *p* = 0.020). Nonetheless, this association did not survive Benjamini–Hochberg false discovery rate correction across the six markers (FDR-adjusted *p* = 0.144) and should therefore be considered a preliminary signal rather than a definitive finding. No significant correlations with ALSFRS–R were observed for the remaining markers, even when potential non-linear dependencies were explored by polynomial regression.

### 3.2. Relationship Between Marker Levels and Disease Duration at the Time of Sampling

#### Interpretation of Results

**Figure 2:**
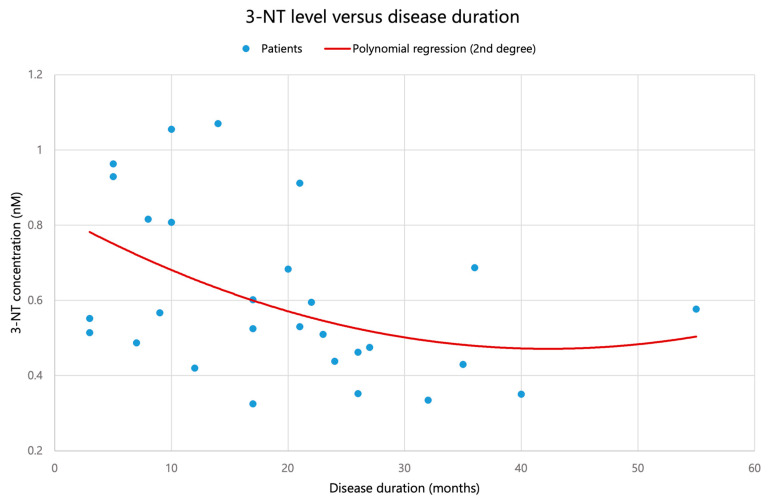
Association between plasma 3–nitrotyrosine (3–NT) concentration and disease duration at sampling in 29 patients with ALS. Points represent individual patients; the displayed trend line is descriptive. After FDR correction, this association did not remain statistically significant.

**Figure 3:**
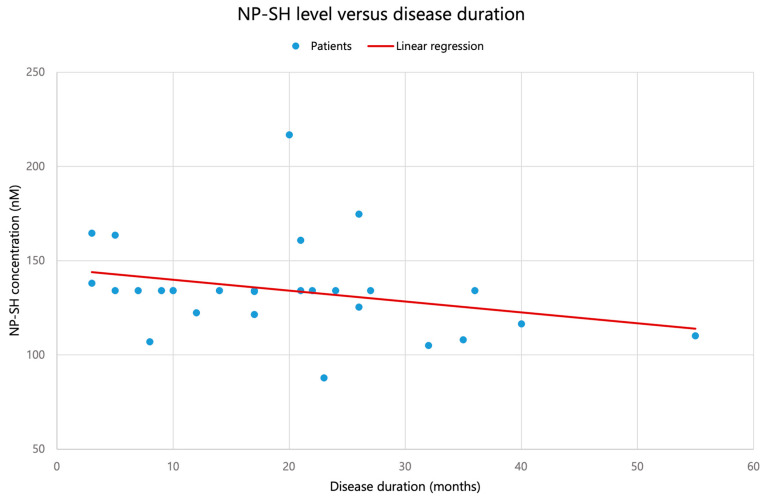
Association between plasma non-protein thiols (NP–SH) and disease duration at sampling in 29 patients with ALS. Points represent individual patients; the displayed trend line is descriptive. After FDR correction, this association did not remain statistically significant.

For disease duration, both 3–NT and NP–SH showed moderate negative monotonic correlations (3–NT: Spearman ρ = −0.438, *p* = 0.020; NP–SH: ρ = −0.404, *p* = 0.033), see Figure 2 and Figure 3. However, these associations did not remain statistically significant after Benjamini–Hochberg correction for multiple testing across the six markers (FDR-adjusted *p* = 0.099 for both 3–NT and NP–SH), as shown in Table 2. Given the limited sample size, these findings should be interpreted as suggestive trends that warrant confirmation in larger cohorts. Importantly, none of the investigated associations remained statistically significant after false discovery rate correction, and all such findings should therefore be interpreted as exploratory.

### 3.3. Conclusions

A moderate, predominantly decreasing association with ALS duration was observed for 3–NT [nM] and NP–SH [µM], with both markers showing negative monotonic correlations with disease duration (3–NT: Spearman ρ = −0.438, *p* = 0.020; NP–SH: ρ = −0.404, *p* = 0.033). However, after Benjamini–Hochberg correction for multiple testing across the six oxidative–nitrosative markers, these associations did not remain statistically significant (FDR-adjusted *p* = 0.099 for both markers) and should therefore be interpreted as suggestive trends rather than definitive findings. No other oxidative–nitrosative stress markers were associated with disease duration in this cohort.

### 3.4. Overall Survival

Survival data were available for the full cohort, with events defined as death (status = 1) and censoring at the last follow-up (status = 0) for patients who were still alive. In a multivariable Cox proportional hazards model including baseline 3–NT, age, sex and BMI, the overall model was statistically significant (likelihood ratio χ^2^ = 12.61, df = 4, *p* = 0.013), indicating that the covariates jointly contributed to the explanation of survival differences, see Table 3.

**Figure 4:**
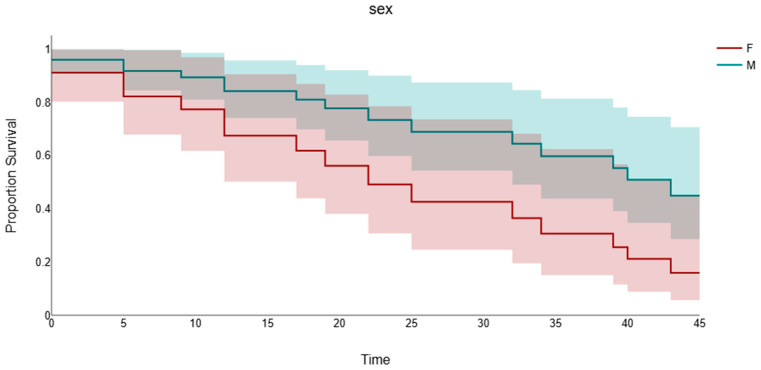
Kaplan–Meier survival curves for patients with amyotrophic lateral sclerosis (ALS) stratified by sex (F = female vs. M = male). Each step represents an event (death), and vertical ticks indicate censored observations at the last follow-up. Survival differences between groups were compared using the log-rank test, and hazard ratios with 95% confidence intervals were additionally estimated from a multivariable Cox proportional hazards model adjusted for age, body mass index (BMI), and baseline 3–nitrotyrosine (3–NT) concentration.

Baseline 3–NT concentration was not significantly associated with overall survival (HR per 1 nM increase 3.44, 95% CI 0.25–47.97, *p* = 0.358), reflecting a highly imprecise estimate with very wide confidence intervals. Age, in contrast, showed a modest but statistically significant association with survival (HR per 1-year increase 1.05, 95% CI 1.00–1.10, *p* = 0.036), whereas sex (female vs. male: HR 2.30, 95% CI 0.47–11.19, *p* = 0.303) and BMI (HR per 1 kg/m^2^ increase 1.12, 95% CI 0.96–1.31, *p* = 0.146) were not significant predictors, see Figure 4. These results suggest that, in this pilot cohort, baseline 3–NT, sex and BMI did not exhibit robust prognostic value for survival, while older age was associated with a higher hazard of death.

#### Secondary Hypotheses

The following secondary hypotheses were evaluated:

- Plasma 3–nitrotyrosine (3–NT) levels positively correlate with urea concentration.
- Plasma 3–NT levels negatively correlate with creatinine concentration.
- Plasma 3–NT levels negatively correlate with uric acid concentration.
- Creatinine concentration positively correlates with ALSFRS–R score.
- Creatinine concentration positively correlates with overall survival.
- Lower uric acid concentration is associated with shorter survival.
- Higher urea concentration is associated with shorter survival.

None of the above hypotheses were statistically confirmed in this cohort. These results may reflect key limitations related to the sample size and the use of a single-time-point sampling design. However, the absence of statistically significant correlations suggests that these individual markers alone are not reliable predictors of functional status or survival in ALS. Their potential prognostic or pathophysiological value should be verified in larger longitudinal studies.

## 4. Discussion

### 4.1. Summary of Main Findings

In this pilot cohort of 29 ALS patients, plasma 3-nitrotyrosine (3–NT) showed a moderate positive correlation with functional status assessed by ALSFRS–R and a moderate negative correlation with disease duration. At the uncorrected significance level, both associations reached statistical significance (ALSFRS–R: Spearman ρ = 0.418, *p* = 0.024; disease duration: ρ = −0.438, *p* = 0.020), and non-protein thiols (NP–SH) also displayed a negative correlation with disease duration (ρ = −0.404, *p* = 0.033).

However, after applying Benjamini–Hochberg false discovery rate correction for multiple testing across the six oxidative–nitrosative markers, these associations no longer met the conventional threshold for statistical significance (FDR-adjusted *p* = 0.144 for 3–NT vs. ALSFRS–R and *p* = 0.099 for both 3–NT and NP–SH vs. disease duration). Although 3–NT and NP–SH showed the most consistent uncorrected trends, no association remained statistically significant after FDR correction, which substantially limits inferential strength and supports the exploratory nature of the study. Within the constraints of the small sample, 3–NT and NP–SH therefore emerge as the markers with the strongest and most consistent trends across clinical outcomes, but their relationships with functional status and disease duration should be regarded as suggestive rather than definitively established. Other markers—8-oxo-2′-deoxyguanosine, MDA, and GSH—showed weak and non-significant correlations with clinical parameters, and none of the analyzed oxidative–nitrosative markers or routine biochemical variables demonstrated robust associations with survival.

In exploratory Cox regression including baseline 3–NT, age, sex and BMI, the overall model reached statistical significance, but 3–NT itself was not associated with survival (HR per 1 nM 3.44, 95% CI 0.25–47.97, *p* = 0.358). The extremely wide confidence interval indicates substantial uncertainty and is consistent with the small sample size and limited number of events. Age emerged as the only significant predictor (HR 1.05 per year, 95% CI 1.00–1.10, *p* = 0.036), whereas sex and BMI showed no statistically significant effects (female vs. male: HR 2.30, 95% CI 0.47–11.19, *p* = 0.303; BMI: HR 1.12 per kg/m^2^, 95% CI 0.96–1.31, *p* = 0.146). Consequently, our data do not support a robust prognostic role for baseline 3–NT in this cohort and underline the need for larger, adequately powered survival studies.

### 4.2. Possible Interpretation of the 3–Nitrotyrosine Findings

The observed positive correlation between plasma 3–NT levels and ALSFRS–R scores, along with its negative relationship with disease duration, suggests that higher 3–NT concentrations are characteristic of the earlier stages of amyotrophic lateral sclerosis. At first glance, this finding may appear paradoxical, as 3–NT is generally considered a product of nitrosative damage, and elevated levels are typically associated with advanced tissue injury. However, in the context of our data, it may be interpreted that 3–NT reflects an active phase of nitrosative stress present in the early stage of the disease, when both the production capacity and structural integrity of tissues are still preserved.

From a biochemical perspective, 3–NT is formed by nitration of the phenolic group of the amino acid tyrosine when it is incorporated into proteins [33]. Tyrosine is considered one of the most reactive proteinogenic amino acids under conditions of oxidative and nitrative stress, reflecting its high susceptibility to modification at the phenolic ring [34]. Tyrosine itself does not react with peroxynitrite directly; rather, peroxynitrite gives rise to secondary radicals, most prominently via its reaction with CO_2_, which oxidize tyrosine to a tyrosyl radical. This tyrosyl radical can then either react with NO_2_^−^ to form 3–NT or dimerize to dityrosine [35,36,37,38]. In addition, transition metals and heme peroxidases (such as myeloperoxidase) can catalyze alternative pathways of NO_2_^−^ formation [39,40,41]. Taken together, 3–NT is therefore best regarded as a marker of overall nitrative stress mediated by reactive nitrogen species rather than a specific footprint of a single oxidant.

In humans, tyrosine is a non-essential amino acid that mainly generated by hydroxylation of the essential amino acid phenylalanine and also obtained from dietary protein, after which it is incorporated into body proteins and serves as a precursor of catecholaminergic neurotransmitters and other bioactive compounds [33,42].

Plasma 3–NT levels may therefore reflect a combination of tyrosine residue availability in tissues and the intensity of nitrosative activity. Based on our findings, two complementary hypotheses can be proposed to explain the observed pattern of changes in 3–NT concentrations over the course of the disease.

Our initial use of second-order polynomial regression was motivated by the biological possibility that oxidative–nitrosative markers might increase in early disease phases and decline later, yielding curved relationships with clinical parameters. However, in this small cohort, the quadratic terms were not robustly significant, adjusted R^2^ values remained modest, and residual diagnostics did not reveal clear evidence of strong non-linearity. To avoid over-interpretation and overfitting, we therefore base our main conclusions on monotonic correlation analyses and treat polynomial models as exploratory tools rather than definitive evidence of complex non-linear patterns.

#### 4.2.1. Hypothesis of Active Nitrosative Stress in the Early Stage of the Disease

This hypothesis assumes that, during the early stages of ALS, an intense phase of nitrosative stress occurs, associated with microglial activation, increased expression of inducible nitric oxide synthase (iNOS), and mitochondrial dysfunction. In this phase, nitric oxide (NO•) and superoxide anion (O_2_•^−^) are simultaneously produced, leading to the formation of peroxynitrite and subsequent extensive protein nitration.

Higher plasma levels of 3–nitrotyrosine (3–NT) may thus reflect an active pathophysiological phase in which nitrative processes are fully underway, but significant tissue loss has not yet occurred. This model supports the concept of oxidative–nitrosative stress acting as an early trigger of neurodegeneration.

#### 4.2.2. Hypothesis of Substrate Depletion and Reduced Production Capacity in the Advanced Stage of the Disease

The second hypothesis explains the decline in 3–nitrotyrosine (3–NT) levels during later stages of ALS because of the depletion of nitration substrates—namely neurons, glial cells, and muscle fibers that contain both tyrosine and systems capable of generating reactive species. As a result, there is a simultaneous reduction in both the available “target” (tyrosine residues) and the “trigger” (peroxynitrite). With progressive atrophy and loss of functional cells, nitration activity and its measurable plasma product, 3–NT, both decrease. This evolution does not necessarily indicate an improvement in oxidative balance but rather a biological exhaustion of substrates and production capacity.

#### 4.2.3. Combination of Two Hypotheses

The two hypotheses are not mutually exclusive but rather complementary. Higher 3–NT levels in the early phase of ALS can be attributed to intense oxidative–nitrosative stress, whereas the subsequent decline likely reflects the gradual loss of the cellular structures required for its generation. This progression corresponds to the temporal dynamics of ALS pathophysiology and supports the concept of 3–NT as a potential early biomarker of nitrosative stress.

#### 4.2.4. Comparison with the Literature

A significant piece of evidence supporting the early onset of nitrosative stress during ALS progression comes from a longitudinal study by Sasaki et al. (2001) [43], who examined the spinal cord of transgenic SOD1-G93A mice throughout the disease course. Using immunohistochemistry, they detected iNOS expression and 3–nitrotyrosine (3–NT) formation at four time points: weeks 24, 28, 32, and 35 of age.

In control animals (wild type), no immunoreactivity for either iNOS or 3–NT was detected in anterior horn motor neurons. In contrast, transgenic mice already showed occasional iNOS and 3–NT positivity in motor neurons and their processes at week 24 (early presymptomatic phase). By week 28 (late presymptomatic phase), the signal was more pronounced, and by weeks 32–35 (early symptomatic and terminal phases), strong immunoreactivity was present in both motor neurons and reactive astrocytes [43].

These data indicate that protein nitration (3–NT formation) occurs prior to the development of clinical symptoms and that its intensity increases as the disease progresses. The detection of 3–NT in relatively preserved tissue supports the hypothesis that nitrosative stress represents an early pathophysiological event in the neurodegenerative process rather than a secondary consequence of advanced tissue destruction.

Similarly, elevated levels of free 3–nitrotyrosine have been demonstrated in the early stages of the disease in the spinal cord of transgenic SOD1-G37R mice, further supporting the role of early nitration in ALS pathogenesis [44].

In a cohort of 19 patients with ALS and 19 controls, cerebrospinal fluid concentrations of 3–NT were significantly higher in sALS, whereas levels of free tyrosine did not differ between groups. Consequently, the 3–nitrotyrosine-to-tyrosine ratio was also significantly elevated in sALS. These markers did not correlate with age, disease duration, or disease severity, suggesting that increased tyrosine nitration represents a disease-related biochemical feature rather than a simple consequence of advancing age or longer disease course. Taken together, these in vivo data are consistent with a contribution of peroxynitrite-mediated oxidative stress and tyrosine nitration to the pathophysiology of sALS, although they do not support a straightforward association with clinical stage or progression rate [45]. Although numerous studies highlight the role of oxidative–nitrosative stress in ALS pathogenesis, none to date have demonstrated a direct relationship between plasma 3–nitrotyrosine levels and clinical parameters such as functional status or disease duration. Most existing studies have focused on post-mortem detection of 3–NT in nervous tissue. Therefore, our results provide the first clinical evidence suggesting the potential utility of 3–nitrotyrosine as a biomarker in living ALS patients.

### 4.3. Significance of the Decrease in NP–SH

Plasma concentrations of non-protein thiols (NP–SH) in this study showed a moderate negative correlation with disease duration, which was statistically significant at the uncorrected level (Spearman ρ = −0.404, *p* = 0.033; Kendall τ = −0.309, *p* = 0.030) but did not remain significant after false discovery rate correction (FDR-adjusted *p* = 0.099). This pattern is consistent with a gradual depletion of systemic antioxidant capacity as the neurodegenerative process advances, although the present evidence should be regarded as exploratory and requires confirmation in larger cohorts.

NP–SH comprise primarily reduced glutathione (GSH), which accounts for more than 90% of all intracellular non-protein thiols [46]. GSH plays a crucial role in maintaining redox homeostasis, neutralizing peroxide radicals via glutathione peroxidases, detoxifying electrophilic compounds through glutathione-S-transferases, and regenerating other antioxidants such as vitamins C and E [47]. It also contributes to preserving the structural integrity of proteins by protecting their thiol groups from oxidation [48].

The observed decrease in NP–SH concentrations can therefore be interpreted as a progressive loss of antioxidant defense during ALS progression. This reduction may result from both increased GSH consumption in neutralizing reactive oxygen species (ROS) and impaired biosynthesis. GSH is synthesized in two enzymatic steps catalyzed by glutamate–cysteine ligase (GCL) and glutathione synthetase (GS), with cysteine serving as the rate-limiting precursor. Because cysteine is highly susceptible to oxidation, its availability may be markedly reduced under chronic oxidative stress [49].

Thus, decreased NP–SH levels in plasma may reflect both enhanced antioxidant utilization and depletion of substrates or enzymatic systems required for GSH regeneration. This observation fits within the broader context of redox imbalance in ALS and reinforces the concept of oxidative stress as a key contributor to disease pathogenesis and progression. Given that NP–SH levels can be measured relatively easily in plasma, they may serve as a practical biomarker of systemic redox status and potentially as a useful indicator of response to antioxidant therapy.

#### Comparison with the Literature

In a longitudinal study investigating neuroinflammation, 13 patients with ALS and 7 healthy controls were examined. ALS patients consistently exhibited markedly reduced serum levels of GSH compared to controls, accompanied by elevated concentrations of proinflammatory cytokines (IL-6, IL-8) and nitrite—a metabolite of nitric oxide (NO) [50]. These findings indicate the presence of systemic oxidative and inflammatory stress in ALS and further support our observation of declining plasma NP–SH levels with increasing disease duration.

A recent study by Grossini et al. evaluated the effects of Acetyl-L-carnitine (ALCAR) on oxidative stress in 32 newly diagnosed ALS patients. At baseline, these patients exhibited elevated levels of TBARS (thiobarbituric acid reactive substances—a marker of lipid peroxidation) and 4-HNE (4-hydroxy-2-nonenal—a toxic product of unsaturated fatty acid oxidation), along with reduced GSH concentrations and lower glutathione peroxidase (GPx) activity compared with healthy controls. After six months of ALCAR treatment, oxidative stress markers decreased, while GSH levels and GPx activity increased. This study underscores the pivotal role of oxidative stress in ALS pathogenesis and suggests that these biochemical parameters could serve as potential indicators of therapeutic response [51].

Our findings, demonstrating a decline in plasma NP–SH (low-molecular-weight thiol groups) in relation to ALS duration, are consistent with these observations and reinforce the hypothesis of a progressive depletion of antioxidant capacity during disease progression.

### 4.4. Study Limitations

This pilot study was conducted on a relatively small cohort of patients, reflecting both the rarity of amyotrophic lateral sclerosis (ALS) and the limited recruitment capacity of our center. Another important limitation is the absence of a healthy or disease control group, which does not allow us to determine whether the observed oxidative–nitrosative stress marker levels are specific to ALS or differ from those in other populations. Consequently, the present study can only describe associations within a small ALS cohort and cannot establish disease specificity or the full biomarker relevance of these markers. Plasma samples were collected at a single time point, so the results capture only cross-sectional relationships between the analyzed parameters and clinical status at a specific moment, rather than their longitudinal evolution throughout the disease course.

We could not fully adjust for potential confounders such as detailed nutritional status or residual variation in renal function beyond creatinine-based assessment, which may influence biomarker levels.

In survival analyses, Kaplan–Meier curves stratified by sex suggested shorter survival in female patients compared with males; however, this visual difference did not reach statistical significance. The multivariable Cox model yielded a non-significant hazard ratio for females versus males (HR 2.30, 95% CI 0.47–11.19, *p* = 0.303), with very wide confidence intervals, underscoring the limited precision of our estimates due to the small sample size and number of events. Consequently, sex-related differences in survival in this cohort should be regarded as hypothesis-generating and require confirmation in larger studies.

### 4.5. Future Research Perspectives

Future studies should aim to validate these findings in a larger cohort of patients and incorporate longitudinal sampling at multiple disease stages. Such an approach would allow tracking the temporal dynamics of biomarker changes, improve understanding of their progression over time, and potentially identify their prognostic or predictive value for clinical practice.

## 5. Conclusions

In this pilot cohort of 29 ALS patients, plasma 3-nitrotyrosine (3–NT) showed a moderate positive correlation with functional status assessed by ALSFRS–R and a negative correlation with disease duration, whereas other oxidative–nitrosative markers and routine biochemical parameters did not demonstrate robust associations with these clinical measures after correction for multiple testing. Non-protein thiols (NP–SH) also declined with longer disease duration, suggesting progressive exhaustion of systemic antioxidant capacity.

Because of the cross-sectional design, limited sample size and absence of longitudinal biomarker measurements, these relationships cannot be interpreted causally, and our mechanistic models of early nitrosative activation and subsequent substrate depletion must be regarded as speculative. Exploratory survival analyses did not reveal significant associations between baseline oxidative–nitrosative markers or routine biochemical parameters and overall survival, with hazard ratios close to 1 and wide confidence intervals.

Taken together, these findings are consistent with a potential involvement of oxidative–nitrosative imbalance, with 3–NT and NP–SH emerging as the most promising candidates; however, the present data do not establish their prognostic value. Larger longitudinal studies with repeated biomarker assessments and adequately powered survival analyses are required to clarify the temporal dynamics and prognostic relevance of these markers in ALS.

## Figures and Tables

**Table 1:** Correlation of selected oxidative and nitrosative stress markers with functional status (ALSFRS–R) in ALS patients.

Marker	N	Spearman ρ	*p* (Uncorrected)	*p* (FDR)	Kendall τ	*p* (Kendall)	Adjusted R^2^
3–nitrotyrosine (3–NT)	29	0.418	0.024	0.144	0.311	0.020	0.161
8-oxo-2′-deoxyguanosine (8-oxoG)	29	−0.083	0.668	0.802	−0.030	0.821	0.062
Malondialdehyde (MDA)	29	0.043	0.825	0.825	0.025	0.850	0.007
Glutathione (GSH)	29	0.272	0.153	0.459	0.205	0.147	0.060
Non-protein thiols (NP–SH)	29	0.165	0.393	0.590	0.127	0.367	0.034
Non-protein disulfides (NP–SS–NP)	29	0.228	0.234	0.468	0.160	0.256	0.202

**Table 2:** Association between disease duration at sampling and plasma oxidative stress markers in ALS patients.

Marker	N	Spearman ρ	*p* (Uncorrected)	*p* (FDR)	Kendall τ	*p* (Kendall)	R^2^ (Polynomial)
3–nitrotyrosine (3–NT)	29	−0.438	0.020	0.099	−0.316	0.020	0.199
8-oxo-2′-deoxyguanosine (8-oxodG)	29	0.272	0.162	0.324	0.179	0.185	0.074
Malondialdehyde (MDA)	29	−0.125	0.527	0.527	−0.099	0.464	0.013
Glutathione (GSH)	29	−0.196	0.318	0.428	−0.138	0.331	0.023
Non-protein thiols (NP–SH)	29	−0.404	0.033	0.099	−0.309	0.030	0.093
Non-protein disulfides (NP–SS–NP)	29	0.179	0.357	0.428	0.129	0.367	0.019

**Table 3:** Multivariable Cox proportional hazards model for overall survival in amyotrophic lateral sclerosis (ALS) patients. The model includes baseline plasma 3-nitrotyrosine (3–NT), age, sex and body mass index (BMI) as covariates.

Marker	Unit	HR (Adjusted)	95% CI	*p* Value
3–nitrotyrosine (3–NT)	per 1 nM	3.44	0.25–47.97	0.358
Age	per 1 year	1.05	1.00–1.10	0.036
Sex (female vs. male)	–	2.30	0.47–11.19	0.303
Body mass index (BMI)	per 1 kg/m^2^	1.12	0.96–1.31	0.146

## Data Availability

The original contributions presented in this study are included in the article/Supplementary Materials. Further inquiries can be directed to the corresponding author.

## Supplementary Materials

The following supporting information can be downloaded at: https://www.mdpi.com/article/10.3390/biom16050721/s1, Dataset S1: Raw data underlying the analyses.

## Abbreviations

The following abbreviations are used in this manuscript:

ALS	amyotrophic lateral sclerosis
ALSFRS–R	Amyotrophic Lateral Sclerosis Functional Rating Scale–Revised
BMI	body mass index
ROS	reactive oxygen species
RNS	reactive nitrogen species
NO-	nitric oxide
O_2_^−•^	superoxide anion
H_2_O_2_	hydrogen peroxide
-OH	hydroxyl radical
ONOO^−^	peroxynitrite
3–NT	3–nitrotyrosine
8–oxodG	8-oxo-2′-deoxyguanosine
MDA	malondialdehyde
GSH	glutathione
NP–SH	non-protein thiols
iNOS	inducible nitric oxide synthase
CoQ10	coenzyme Q10
PUA	plasma uric acid
PCr	plasma creatinine
IsoP	15-F_2_t-isoprostane
CK	creatine kinase
TBARS	thiobarbituric acid reactive substances
4-HNE	4-hydroxy-2-nonenal
GPx/GSH-Px	glutathione peroxidase
SOD/Cu,Zn-SOD	superoxide dismutase/copper-zinc superoxide dismutase
NP–SS–NP	non-protein disulfides
DNPH	2,4-dinitrophenylhydrazine
SPE	solid-phase extraction
HPLC-MS/MS	high-performance liquid chromatography coupled with tandem mass spectrometry
APCI	atmospheric pressure chemical ionization
HPLC-UV	high-performance liquid chromatography with ultraviolet detection
DTNB	5,5′-dithiobis-(2-nitrobenzoic) acid (Ellman’s reagent)
NaBH_4_	sodium borohydride
UHPLC-MS/MS	ultra-high-performance liquid chromatography coupled with tandem mass spectrometry
ESI	electrospray ionization
SRM	selected reaction monitoring
R^2^	coefficient of determination
Spearman’s rho	Spearman’s rank correlation coefficient
Kendall τ	Kendall’s tau
